# 4-[1-(Hydroxy­imino)ethyl]-*N*-(4-nitro­benzyl­idene)aniline

**DOI:** 10.1107/S1600536809033753

**Published:** 2009-09-12

**Authors:** Li Zhao, Wen-Kui Dong, Jian-Chao Wu, Yin-Xia Sun, Li Xu

**Affiliations:** a School of Chemical and Biological Engineering, Lanzhou Jiaotong University, Lanzhou 730070, People’s Republic of China

## Abstract

In the title compound, C_15_H_13_N_3_O_3_, the dihedral angle formed by the two benzene rings is 44.23 (2)°. The crystal structure is stabilized by aromatic π–π stacking inter­actions, with centroid-centroid distances of 3.825 (3) and 3.870 (4) Å between the aniline and the nitro­benzene rings of neighbouring mol­ecules, respectively. In addition, the stacked mol­ecules exhibit inter­molecular C—H⋯N and C—H⋯O inter­actions.

## Related literature

For background to Schiff bases, see: Lozier *et al.* (1975 ▶). For the synthesis, see: Rafiq *et al.* (2008 ▶); Duan *et al.* (2007 ▶); Dong *et al.* (2008 ▶). For related structures, see: Bomfim *et al.* (2005 ▶); Fun *et al.* (2008 ▶).
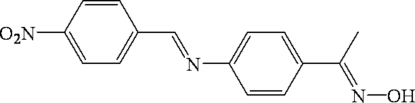


## Experimental

### 

#### Crystal data

C_15_H_13_N_3_O_3_
*M* *_r_* = 283.28
Orthorhombic, 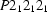

*a* = 7.375 (1) Å
*b* = 10.770 (2) Å
*c* = 16.906 (2) Å
*V* = 1342.8 (3) Å^3^
*Z* = 4
Mo *K*α radiation
μ = 0.10 mm^−1^
*T* = 298 K
0.50 × 0.35 × 0.10 mm

#### Data collection

Bruker SMART1000 CCD area-detector diffractometer
Absorption correction: multi-scan (*SADABS*; Sheldrick, 1996 ▶) *T*_min_ = 0.952, *T*_max_ = 0.990
7656 measured reflections
1700 independent reflections
899 reflections with *I* > 2σ(*I*)
*R*_int_ = 0.068

#### Refinement

*R*[*F* ^2^ > 2σ(*F* ^2^)] = 0.043
*wR*(*F* ^2^) = 0.115
*S* = 1.03
1700 reflections
195 parameters
2 restraints
H atoms treated by a mixture of independent and constrained refinement
Δρ_max_ = 0.20 e Å^−3^
Δρ_min_ = −0.17 e Å^−3^

### 

Data collection: *SMART* (Siemens, 1996 ▶); cell refinement: *SAINT* (Siemens, 1996 ▶); data reduction: *SAINT*; program(s) used to solve structure: *SHELXS97* (Sheldrick, 2008 ▶); program(s) used to refine structure: *SHELXL97* (Sheldrick, 2008 ▶); molecular graphics: *SHELXTL* (Sheldrick, 2008 ▶); software used to prepare material for publication: *SHELXTL*.

## Supplementary Material

Crystal structure: contains datablocks 2, I. DOI: 10.1107/S1600536809033753/lx2109sup1.cif

Structure factors: contains datablocks I. DOI: 10.1107/S1600536809033753/lx2109Isup2.hkl

Additional supplementary materials:  crystallographic information; 3D view; checkCIF report

## Figures and Tables

**Table 1:** Hydrogen-bond geometry (Å, °)

*D*—H⋯*A*	*D*—H	H⋯*A*	*D*⋯*A*	*D*—H⋯*A*
O1—H1⋯N2^i^	0.95 (4)	1.97 (4)	2.887 (4)	162 (4)
C1—H1*A*⋯O3^ii^	0.96	2.62	3.469 (5)	148

Symmetry codes: (i) 
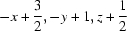
; (ii) 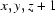.

**Fig. 1.**
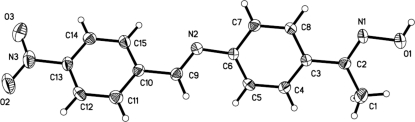
The molecule structure of the title compound with atom numbering. Displacement ellipsoids for non-hydrogen atoms are drawn at the 30% probability level.

**Fig. 2.**
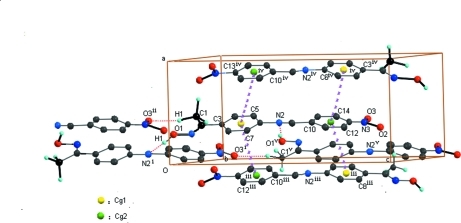
π···π, O—H···N and C—H···O interactions (dotted lines) in the crystal structure of the title compound. Cg denotes the ring centroids. [Symmetry code: (i) -x + 3/2, -y + 1, z + 1/2; (ii) x, y, z + 1; (iii) x + 1/2, -y + 3/2, -z + 1; (iv) x - 1/2, -y + 3/2, -z + 1; (v) -x + 3/2, -y + 1, z - 1/2.]

## supplementary crystallographic information

### Comment

It is well known that Schiff bases are one of the most popular mixed-donor ligands in the field of coordination chemistry. Schiff bases often exhibit various biological activities and in many cases were shown to have antibacterial, anticancer, anti-inflammatory and antitoxic properties (Lozier *et al.*, 1975). Some structures of oxime compounds forming by Schiff bases reaction have been reported (Bomfim *et al.*, 2005; Fun *et al.*, 2008). Here we report the synthesis and crystal structure of the title compound (I), (Fig. 1).

The dihedral angle in (I) formed by the aniline and nitrobenzene rings is 44.23 (2)°. The molecular packing (Fig. 2) is stabilized by aromatic π···π interactions between the aniline and the nitrobenzene rings of neighbouring molecules, with a *Cg*1···*Cg*2^iii^ separation of 3.825 (3) Å and a *Cg*1···*Cg*2^iv^ separation of 3.870 (4) Å (Fig. 2; *Cg*1 and *Cg*2 are the centroids of the C3—C8 benzene and the C10–C15 benzene rings, respectively). Additionally, intermolecular O–H···N and C—H···O interactions in the structure were observed (Table 1 and Fig. 2).

The title compound is not chiral, but space group is p212121. This is because the title compound is rigid in the crystal, and adopts a chiral helicalx-type structure.

### Experimental

4-Aminophenylethanone oxime was prepared by 1-(4-aminophenyl)ethanone, hydroxylamine sulfate and sodium acetate (Rafiq *et al.*, 2008; Duan *et al.*, 2007; Dong *et al.*, 2008). To an ethanol solution (5 ml) of 4-aminophenylethanone oxime (150.2 mg, 1.00 mmol) was added dropwise an ethanol solution (5 ml) of 4-nitrobenzaldehyde (152.5 mg, 1.01 mmol). The mixture solution was stirred at 328–333 K for 5 h. After cooling to room temperature, the precipitate was filtered off, and washed successively three times with ethanol. The product was dried *in vacuo* and purified by recrystallization from ethanol to yield 367.5 mg (Yield, 82.6%) of solid; m.p. 484–485 K. Pale-yellow block-like single crystals suitable for X-ray diffraction studies were obtained by slow evaporation from a solution of ethyl acetate of (I) at room temperature for about one month. Anal. Calcd. for C_15_H_13_N_3_O_3_: C, 62.6; H, 4.63; N, 14.83 Found: C, 62.1; H, 4.59; N, 14.87.

### Refinement

Atom H1 of the hydroxy group was found in a difference Fourier map and was refined with an O—H distance restraint of 0.95 (4) Å. The other H atoms were treated as riding atoms with distances C—H = 0.96 (CH_3_), 0.93 Å (CH), and *U*_iso_(H) = 1.2 *U*_eq_(C) and 1.5 *U*_eq_(O). In the absence of significant anomalous scattering effects, Friedel pairs were merged.

### Crystal data

**Table:**

C_15_H_13_N_3_O_3_	*F*(000) = 592
*M**_r_* = 283.28	*D*_x_ = 1.401 Mg m^−^^3^
Orthorhombic, *P*2_1_2_1_2_1_	Mo *K*α radiation, λ = 0.71073 Å
Hall symbol: P 2ac 2ab	Cell parameters from 1209 reflections
*a* = 7.375 (1) Å	θ = 2.2–21.4°
*b* = 10.770 (2) Å	µ = 0.10 mm^−^^1^
*c* = 16.906 (2) Å	*T* = 298 K
*V* = 1342.8 (3) Å^3^	Block-like, pale-yellow
*Z* = 4	0.50 × 0.35 × 0.10 mm

### Data collection

**Table:**

Bruker SMART1000 CCD area-detector diffractometer	1700 independent reflections
Radiation source: fine-focus sealed tube	899 reflections with *I* > 2σ(*I*)
graphite	*R*_int_ = 0.068
phi and ω scans	θ_max_ = 27.0°, θ_min_ = 2.2°
Absorption correction: multi-scan (*SADABS*; Sheldrick, 1996)	*h* = −9→9
*T*_min_ = 0.952, *T*_max_ = 0.990	*k* = −13→13
7656 measured reflections	*l* = −14→21

### Refinement

**Table:**

Refinement on *F*^2^	Primary atom site location: structure-invariant direct methods
Least-squares matrix: full	Secondary atom site location: difference Fourier map
*R*[*F*^2^ > 2σ(*F*^2^)] = 0.043	Hydrogen site location: inferred from neighbouring sites
*wR*(*F*^2^) = 0.115	H atoms treated by a mixture of independent and constrained refinement
*S* = 1.03	*w* = 1/[σ^2^(*F*_o_^2^) + (0.0438*P*)^2^ + 0.0834*P*] where *P* = (*F*_o_^2^ + 2*F*_c_^2^)/3
1700 reflections	(Δ/σ)_max_ < 0.001
195 parameters	Δρ_max_ = 0.20 e Å^−^^3^
2 restraints	Δρ_min_ = −0.17 e Å^−^^3^

### Special details

**Table:**

Geometry. All e.s.d.'s (except the e.s.d. in the dihedral angle between two l.s. planes) are estimated using the full covariance matrix. The cell e.s.d.'s are taken into account individually in the estimation of e.s.d.'s in distances, angles and torsion angles; correlations between e.s.d.'s in cell parameters are only used when they are defined by crystal symmetry. An approximate (isotropic) treatment of cell e.s.d.'s is used for estimating e.s.d.'s involving l.s. planes.
Refinement. Refinement of *F*^2^ against ALL reflections. The weighted *R*-factor *wR* and goodness of fit *S* are based on *F*^2^, conventional *R*-factors *R* are based on *F*, with *F* set to zero for negative *F*^2^. The threshold expression of *F*^2^ > σ(*F*^2^) is used only for calculating *R*-factors(gt) *etc*. and is not relevant to the choice of reflections for refinement. *R*-factors based on *F*^2^ are statistically about twice as large as those based on *F*, and *R*- factors based on ALL data will be even larger.

### Fractional atomic coordinates and isotropic or equivalent isotropic displacement parameters (Å^2^)

**Table:**

	*x*	*y*	*z*	*U*_iso_*/*U*_eq_	
N1	0.7084 (5)	0.5375 (3)	0.90756 (16)	0.0478 (9)	
N2	0.6140 (4)	0.6960 (3)	0.54920 (16)	0.0389 (8)	
N3	0.6281 (5)	0.8627 (4)	0.1831 (2)	0.0601 (11)	
O1	0.7150 (4)	0.5267 (3)	0.99032 (15)	0.0636 (10)	
H1	0.784 (6)	0.454 (4)	0.999 (3)	0.100 (18)*	
O2	0.6733 (5)	0.9635 (3)	0.15589 (17)	0.0830 (11)	
O3	0.5894 (6)	0.7723 (3)	0.14311 (17)	0.0939 (13)	
C1	0.5534 (7)	0.7318 (4)	0.9402 (2)	0.0755 (15)	
H1A	0.5821	0.7090	0.9936	0.113*	
H1B	0.4241	0.7352	0.9339	0.113*	
H1C	0.6046	0.8117	0.9286	0.113*	
C2	0.6304 (5)	0.6371 (3)	0.8846 (2)	0.0384 (9)	
C3	0.6214 (5)	0.6542 (3)	0.79802 (19)	0.0319 (9)	
C4	0.5531 (5)	0.7611 (3)	0.76460 (19)	0.0394 (10)	
H4	0.5084	0.8235	0.7972	0.047*	
C5	0.5498 (5)	0.7776 (3)	0.6832 (2)	0.0391 (10)	
H5	0.5041	0.8510	0.6621	0.047*	
C6	0.6133 (5)	0.6869 (3)	0.63323 (19)	0.0328 (9)	
C7	0.6787 (5)	0.5777 (3)	0.6663 (2)	0.0381 (10)	
H7	0.7201	0.5145	0.6334	0.046*	
C8	0.6832 (5)	0.5618 (3)	0.7469 (2)	0.0377 (9)	
H8	0.7283	0.4880	0.7677	0.045*	
C9	0.6249 (5)	0.8019 (3)	0.5170 (2)	0.0402 (10)	
H9	0.6364	0.8719	0.5489	0.048*	
C10	0.6198 (5)	0.8171 (3)	0.43071 (19)	0.0375 (10)	
C11	0.6747 (5)	0.9298 (3)	0.3977 (2)	0.0454 (11)	
H11	0.7098	0.9948	0.4305	0.055*	
C12	0.6773 (5)	0.9453 (4)	0.3167 (2)	0.0466 (11)	
H12	0.7156	1.0198	0.2944	0.056*	
C13	0.6223 (5)	0.8484 (4)	0.2699 (2)	0.0407 (10)	
C14	0.5622 (5)	0.7378 (3)	0.3007 (2)	0.0436 (11)	
H14	0.5223	0.6743	0.2678	0.052*	
C15	0.5627 (5)	0.7232 (3)	0.3816 (2)	0.0405 (10)	
H15	0.5237	0.6485	0.4034	0.049*	

### Atomic displacement parameters (Å^2^)

**Table:**

	*U*^11^	*U*^22^	*U*^33^	*U*^12^	*U*^13^	*U*^23^
N1	0.069 (3)	0.0466 (19)	0.0279 (16)	0.0068 (19)	−0.0017 (17)	0.0065 (15)
N2	0.043 (2)	0.0377 (17)	0.0363 (18)	0.0016 (17)	0.0005 (16)	0.0021 (14)
N3	0.060 (3)	0.080 (3)	0.040 (2)	0.017 (3)	0.003 (2)	0.013 (2)
O1	0.097 (3)	0.0590 (19)	0.0348 (15)	0.014 (2)	−0.0051 (16)	0.0064 (14)
O2	0.100 (3)	0.094 (2)	0.0551 (19)	0.010 (2)	0.0065 (19)	0.0338 (19)
O3	0.139 (4)	0.100 (3)	0.0422 (19)	0.005 (3)	−0.002 (2)	−0.0081 (19)
C1	0.111 (4)	0.072 (3)	0.044 (2)	0.031 (3)	−0.003 (3)	−0.011 (2)
C2	0.043 (3)	0.034 (2)	0.038 (2)	−0.007 (2)	0.0012 (18)	−0.0009 (17)
C3	0.030 (2)	0.034 (2)	0.032 (2)	−0.0030 (19)	−0.0013 (17)	0.0024 (16)
C4	0.042 (3)	0.038 (2)	0.038 (2)	0.002 (2)	0.0005 (18)	−0.0033 (18)
C5	0.042 (3)	0.033 (2)	0.043 (2)	0.0047 (19)	−0.0014 (18)	0.0071 (17)
C6	0.034 (2)	0.037 (2)	0.027 (2)	−0.0009 (19)	0.0004 (17)	0.0015 (15)
C7	0.042 (3)	0.035 (2)	0.037 (2)	−0.001 (2)	0.0061 (19)	−0.0006 (17)
C8	0.043 (3)	0.0325 (19)	0.037 (2)	0.002 (2)	−0.0015 (18)	0.0024 (17)
C9	0.044 (3)	0.041 (2)	0.035 (2)	0.000 (2)	−0.0051 (19)	−0.0031 (17)
C10	0.038 (3)	0.039 (2)	0.035 (2)	0.0029 (19)	0.0034 (19)	−0.0022 (17)
C11	0.045 (3)	0.042 (2)	0.049 (2)	0.002 (2)	−0.005 (2)	0.0022 (18)
C12	0.048 (3)	0.042 (2)	0.050 (3)	0.004 (2)	0.000 (2)	0.0166 (19)
C13	0.039 (3)	0.051 (2)	0.032 (2)	0.013 (2)	0.0026 (18)	0.0079 (18)
C14	0.043 (3)	0.044 (2)	0.044 (2)	0.008 (2)	−0.0051 (18)	−0.0009 (19)
C15	0.039 (3)	0.042 (2)	0.041 (2)	0.003 (2)	0.0038 (18)	0.0064 (18)

### Geometric parameters (Å, °)

**Table:**

N1—C2	1.278 (4)	C5—H5	0.9300
N1—O1	1.405 (4)	C6—C7	1.388 (4)
N2—C9	1.266 (4)	C7—C8	1.375 (5)
N2—C6	1.424 (4)	C7—H7	0.9300
N3—O3	1.219 (4)	C8—H8	0.9300
N3—O2	1.225 (4)	C9—C10	1.469 (5)
N3—C13	1.476 (5)	C9—H9	0.9300
O1—H1	0.95 (4)	C10—C15	1.375 (5)
C1—C2	1.498 (5)	C10—C11	1.396 (5)
C1—H1A	0.9600	C11—C12	1.378 (5)
C1—H1B	0.9600	C11—H11	0.9300
C1—H1C	0.9600	C12—C13	1.371 (5)
C2—C3	1.477 (4)	C12—H12	0.9300
C3—C4	1.378 (4)	C13—C14	1.374 (5)
C3—C8	1.395 (4)	C14—C15	1.376 (4)
C4—C5	1.388 (4)	C14—H14	0.9300
C4—H4	0.9300	C15—H15	0.9300
C5—C6	1.374 (5)		
			
C2—N1—O1	112.8 (3)	C8—C7—C6	120.9 (3)
C9—N2—C6	119.4 (3)	C8—C7—H7	119.6
O3—N3—O2	124.2 (4)	C6—C7—H7	119.6
O3—N3—C13	117.5 (4)	C7—C8—C3	121.1 (3)
O2—N3—C13	118.3 (4)	C7—C8—H8	119.4
N1—O1—H1	104 (3)	C3—C8—H8	119.4
C2—C1—H1A	109.5	N2—C9—C10	121.7 (3)
C2—C1—H1B	109.5	N2—C9—H9	119.1
H1A—C1—H1B	109.5	C10—C9—H9	119.1
C2—C1—H1C	109.5	C15—C10—C11	119.1 (3)
H1A—C1—H1C	109.5	C15—C10—C9	121.7 (3)
H1B—C1—H1C	109.5	C11—C10—C9	119.2 (3)
N1—C2—C3	115.2 (3)	C12—C11—C10	120.4 (4)
N1—C2—C1	123.5 (3)	C12—C11—H11	119.8
C3—C2—C1	121.3 (3)	C10—C11—H11	119.8
C4—C3—C8	117.5 (3)	C13—C12—C11	118.5 (4)
C4—C3—C2	121.8 (3)	C13—C12—H12	120.8
C8—C3—C2	120.7 (3)	C11—C12—H12	120.8
C3—C4—C5	121.3 (3)	C12—C13—C14	122.4 (3)
C3—C4—H4	119.3	C12—C13—N3	119.1 (4)
C5—C4—H4	119.3	C14—C13—N3	118.5 (4)
C6—C5—C4	120.8 (3)	C13—C14—C15	118.4 (4)
C6—C5—H5	119.6	C13—C14—H14	120.8
C4—C5—H5	119.6	C15—C14—H14	120.8
C5—C6—C7	118.3 (3)	C10—C15—C14	121.1 (4)
C5—C6—N2	124.4 (3)	C10—C15—H15	119.5
C7—C6—N2	117.3 (3)	C14—C15—H15	119.5
			
O1—N1—C2—C3	179.3 (3)	C6—N2—C9—C10	−177.8 (3)
O1—N1—C2—C1	−0.3 (6)	N2—C9—C10—C15	15.0 (6)
N1—C2—C3—C4	−175.2 (3)	N2—C9—C10—C11	−164.9 (4)
C1—C2—C3—C4	4.4 (6)	C15—C10—C11—C12	−2.1 (6)
N1—C2—C3—C8	4.4 (5)	C9—C10—C11—C12	177.8 (3)
C1—C2—C3—C8	−175.9 (4)	C10—C11—C12—C13	1.0 (6)
C8—C3—C4—C5	−1.5 (5)	C11—C12—C13—C14	1.0 (6)
C2—C3—C4—C5	178.2 (4)	C11—C12—C13—N3	−178.7 (4)
C3—C4—C5—C6	0.6 (6)	O3—N3—C13—C12	175.4 (4)
C4—C5—C6—C7	0.8 (6)	O2—N3—C13—C12	−3.7 (6)
C4—C5—C6—N2	179.7 (3)	O3—N3—C13—C14	−4.3 (6)
C9—N2—C6—C5	28.3 (6)	O2—N3—C13—C14	176.5 (4)
C9—N2—C6—C7	−152.8 (4)	C12—C13—C14—C15	−1.9 (6)
C5—C6—C7—C8	−1.3 (6)	N3—C13—C14—C15	177.8 (4)
N2—C6—C7—C8	179.8 (3)	C11—C10—C15—C14	1.3 (6)
C6—C7—C8—C3	0.4 (6)	C9—C10—C15—C14	−178.6 (3)
C4—C3—C8—C7	1.0 (6)	C13—C14—C15—C10	0.7 (6)
C2—C3—C8—C7	−178.7 (3)		

### Hydrogen-bond geometry (Å, °)

**Table:**

*D*—H···*A*	*D*—H	H···*A*	*D*···*A*	*D*—H···*A*
O1—H1···N2^i^	0.95 (4)	1.97 (4)	2.887 (4)	162 (4)
C1—H1A···O3^ii^	0.96	2.62	3.469 (5)	148

Symmetry codes: (i) −*x*+3/2, −*y*+1, *z*+1/2; (ii) *x*, *y*, *z*+1.
